# LncRNA IGFBP4-1 promotes tumor development by activating Janus kinase-signal transducer and activator of transcription pathway in bladder urothelial carcinoma: retraction

**DOI:** 10.7150/ijbs.89601

**Published:** 2023-09-07

**Authors:** Chunjing Li, Yu Cao, Li Zhang, Jierong Li, Huayan Wu, Fengsheng Ling, Jintao Zheng, Jianfeng Wang, Bowei Li, Jun He, Xumin Xie, Zhilin Li, Yiping Chen, Xuemei He, Mingjuan Guo, Huiling Wei, Jing Ye, Yun Guo, Shilin Zhang, Liang Liu, Guoqing Liu, Chunxiao Liu

**Affiliations:** 1 Department of Urology, Affiliated Foshan Maternal and Child Healthcare Hospital, Southern Medical University, Foshan, China.; 2 The second school of Clinical Medicine, Southern Medical University, Foshan, China.; 3 Ningxiang People's Hospital, The Affiliated Hospital of Hunan University of Traditional Chinese Medicine, Ningxiang, China.; 4 Department of Urology, Zhujiang Hospital of Southern Medical University, Guangzhou, China.

The authors decided to retract this article, since some authors did not know the publication of the paper and the first author also disagreed with his personal ranking in the paper. The authors take full responsibility for these oversights, and apologize for any confusion or inconvenience that this may have caused to the readers, the journal, and the scientific community as a whole.

